# Endoplasmic reticulum quality control of LDLR variants associated with familial hypercholesterolemia

**DOI:** 10.1002/2211-5463.12740

**Published:** 2019-10-23

**Authors:** Praseetha Kizhakkedath, Anne John, Buthaina K. Al‐Sawafi, Lihadh Al‐Gazali, Bassam R. Ali

**Affiliations:** ^1^ Department of Pathology College of Medicine and Health Sciences United Arab Emirates University Al‐Ain United Arab Emirates; ^2^ Department of Paediatrics College of Medicine and Health Sciences United Arab Emirates University Al‐Ain United Arab Emirates; ^3^ Zayed Center for Health Sciences United Arab Emirates University Al‐Ain United Arab Emirates

**Keywords:** ERAD, FH, LDLR, missense mutation, VLDLR

## Abstract

Loss‐of‐function mutations in the low‐density lipoprotein receptor (*LDLR*) gene can cause familial hypercholesterolemia (FH), but detailed functional evidence for pathogenicity is limited to a few reported mutations. Here, we investigated the cellular pathogenic mechanisms of three mutations in LDLR causing FH, which are structurally identical to pathogenic mutations in the very low‐density lipoprotein receptor (VLDLR). Similar to the VLDLR mutants, LDLR mutants D482H and C667F were found to be localized to the ER, while D445E, which is a conserved amino acid change, did not affect the trafficking of the receptor to the plasma membrane, as confirmed by the N‐glycosylation profile. Although the ER‐retained mutant proteins were soluble, induction of ER stress was observed as indicated by spliced X‐box binding protein‐1 (XBP‐1) mRNA levels. The mutants were found to associate with ER quality control components, and their stability was enhanced by inhibitors of proteasome. Our results contribute to the growing list of transport‐deficient class II LDLR variants leading to FH and provide evidence for the involvement of endoplasmic reticulum‐associated degradation in their stability.

**Abbreviations**
CAD: coronary artery disease
DSP: dithiobis succinimidyl propionate
ER: endoplasmic reticulum
ERAD: endoplasmic reticulum‐associated degradation
FH: familial hypercholesterolemia
LDLR: low‐density lipoprotein receptor
VLDLR: very low‐density lipoprotein receptor
XBP‐1: X‐box binding protein‐1

Familial hypercholesterolemia (FH) is an inherited disorder characterized by elevated serum low‐density lipoprotein (LDL) cholesterol levels, deposition of excess cholesterol in tissues, and premature symptoms of coronary artery disease (CAD) 1. The disease is inherited in an autosomal dominant manner, and homozygotes exhibit a more severe phenotype than heterozygotes. FH heterozygotes account for 1/20 of those presenting with early CAD and ~ 5% of myocardial infarctions (MIs) in persons below 60 years of age 2. It has been suggested that the prevalence of FH is 1 in 230–250 individuals and that < 1% of FH patients have been identified across the globe 3, 4. FH heterozygote plasma cholesterol levels are twice as high as normal, resulting in distinctive cholesterol deposits (xanthomas) in tendons and skin. Approximately 75% of male FH heterozygotes develop CAD, and 50% have a fatal MI by the age of 60 years. In women, the equivalent figures are 45% and 15% 2. Familial hypercholesterolemia, 1 (FHCL1, OMIM#143890) results from mutations occurring in the low‐density lipoprotein receptor (*LDLR*) and is the most prevalent form of autosomal dominant hypercholesterolemia 5, 6. Other forms of monogenic FH are FHCL2 (OMIM#144010), caused by mutation in the apolipoprotein B (*APOB*) gene and FHCL3 (OMIM#603776) caused by mutation in the proprotein convertase subtilisin/kexin type 9 (*PCSK9*) gene 5, 6.

Cholesterol is a component of the plasma membrane, which can be derived either by endogenous intracellular synthesis or by uptake via LDL receptors on their external surfaces. Newly synthesized receptor protein is glycosylated in the Golgi apparatus before passing to the plasma membrane, where it becomes localized in coated pits lined with the protein clathrin. LDL‐bound cholesterol attaches to the receptor and the coated pit sinks inwards, internalizing the LDL particle. There the lipid separates from the receptor and inhibits de novo cholesterol synthesis. The receptor then returns to bind another LDL on the surface. Each LDLR repeats this cycle every 10 min 1. High cholesterol levels in the circulation of FH heterozygotes arise from defective LDLRs. There are over 900 FH alleles in five classes: class I: no LDLR protein produced, class II: LDLR synthesis fails before glycosylation preventing plasma membrane transport, class III: glycosylated LDLR reaches the coated pits, but cannot bind LDL, class IV: receptors reach the plasma membrane but fail to congregate in coated pits, and class V: the receptor cannot release bound LDL 7.

Missense mutations that generate trafficking‐defective variants of LDLR (class II) have been reported to be sequestered in endoplasmic reticulum (ER) and degraded by ubiquitin‐proteasome‐mediated pathway 8. Protein folding in the ER is an inherently error‐prone process, and many mutations increase the chance of protein misfolding in the ER. The misfolded proteins are retained in the ER to facilitate folding, and terminally misfolded proteins subsequently become substrates for ER‐associated degradation (ERAD), a collective process for quality control in the ER 9. In mammals, the ubiquitin ligase HRD1 along with its partner SEL1L acts as a hub that coordinates substrate recognition, ubiquitination, extraction to the cytoplasm via recruitment of AAA ATPase p97/VCP, and subsequent delivery to the proteasome 10, 11. ERAD and its dysfunction have been linked to several human diseases 12. Inactivation of ERAD exerts stress in the ER by the accumulation of misfolded proteins and induces a stress‐responsive program called the unfolded protein response pathway (UPR) 13. In mammals, inositol‐requiring enzyme 1α is the most conserved stress sensor, which when activated, catalyzes the splicing of the mRNA of the transcription factor X‐box binding protein‐1 (XBP‐1) that targets expression of genes to alleviate ER stress 13. Some class II LDLR mutants associated with FH have been reported to induce ER stress and activation of UPR pathways 14.

Three missense mutations (c.1459G>T; p.D487Y, c.1561G>C; p.D521H and c.2117G>T; p.C706F), occurring in a receptor closely related to LDLR, the VLDL receptor (VLDLR), have been previously described to be associated with a rare genetic condition termed dysequilibrium syndrome (DES) 15, 16, 17. The three residues (D487, D521, and C706) mutated in VLDLR in DES are also conserved in the LDLR (D445, D482, C667). We have reported previously that the pathogenic missense mutations in VLDLR result in the ER retention and loss‐of‐function of the mutants 18. The corresponding VLDLR mutants were found to be aggregation‐prone and found to exert ER stress and are degraded by the ubiquitin‐proteasome pathway 19. Mutations affecting the corresponding amino acid residues in LDLR (D445E, D482H, and C667F) have been reported to be pathogenic FH variants. The D445E mutation has been reported in two publications in two patients 20, 21, and one of the patients had serum LDL cholesterol level of 15.9 mm 20. No other missense mutations have been reported at this position. Various pathogenic substitutions have been reported at amino acid residue D482, and the D482H mutation has been reported in three patients 22, 23. All the patients were reported to have fulfilled the clinical diagnostic criteria for FH. So far, only one patient with the C667F mutation has been reported 23 in a patient fulfilling clinical diagnosis criteria of > 6.7 mm serum LDL cholesterol, though other pathogenic missense mutations have been reported multiple times at this locus. However, their cellular mechanisms and degradation have not been studied in detail yet.

In this report, we aimed to delineate the cellular effects of mutations in LDLR by a combination of confocal microscopy and biochemical methods. Our results indicate that mutations leading to critical amino acid changes at conserved positions on residues D482 and C667 resulted in defective trafficking of the LDLR receptor. Mutation at a critical residue causing a conserved amino acid change (D445E) did not affect the cell surface trafficking of the LDLR suggesting another pathogenic mechanism or a nonpathogenic variant. We also report that ER‐retained LDLR mutants engage in interactions with folding chaperones and known ERAD factors and are subjected to proteasomal degradation.

## Materials and methods

### Antibodies

The antibodies with their dilutions and sources were as follows: antibodies for immunofluorescence: mouse monoclonal anti‐HA‐tag [1 : 200; Cell Signaling Technologies (CST; Danvers, MA, USA)], rabbit polyclonal anti‐calnexin (CANX, 1 : 200; Santa Cruz Biotechnology, Dallas, TX, USA), Alexa Fluor 568‐goat anti‐mouse IgG (1 : 200; Molecular Probes, Eugene, OR, USA), Alexa Fluor 647‐goat anti‐rabbit IgG (1 : 200; Molecular Probes). Antibodies for western blotting: rabbit polyclonal anti‐HA (1 : 4000; Sigma‐Aldrich, St. Louis, MO, USA), anti‐Histone H3(1 : 1000, CST), rabbit polyclonal anti‐GAPDH (1 : 2500; Abcam, Cambridge, UK), mouse monoclonal anti‐α‐tubulin (1 : 10 000; Sigma‐Aldrich), rabbit anti‐ CANX (1 : 1000; CST), rabbit anti‐BiP (1 : 1000, CST), rabbit anti‐GRP94 (1 : 1000, CST), mouse anti‐ERP72 (1 : 200; Santa Cruz Biotechnology), goat anti‐SEL1L (1 : 200; Santa Cruz Biotechnology), rabbit anti‐HRD1 (1 : 500; CST), rabbit anti‐OS‐9 (1 : 500; Abcam), goat anti‐rabbit IgG‐peroxidase (1 : 50 000; Sigma‐Aldrich), and rabbit anti‐mouse IgG‐peroxidase (1 : 80 000; Sigma‐Aldrich).

### Generation of mutant expression constructs

All the mutations described in this study are with reference to the coding LDLR sequence represented by GenBank accession number NM_000527.4. A C‐terminal HA epitope tag was introduced to the wild‐type LDLR ORF cloned in plasmid pC3‐LDLR (a kind gift from Al‐Allaf, Imperial College London) by sequential site‐directed mutagenesis. Sequences of all the primers used in this study are listed in Table 1. The primer pairs LDLR_HA1F and LDLR_HA1R were used for introducing the first five amino acid codons of HA‐tag, and the primer pair LDLR_HA2F and LDLR_HA2R were used for introducing the remaining four amino acid codons of the HA‐tag. The missense mutations (D445E: C>G, D482H: G>C, C>T, C667F: G>T) were introduced into the LDLR‐HA expression vector by site‐directed mutagenesis using PfuUltra HF polymerase (Stratagene, La Jolla, CA, USA). The primers for introducing the mutations are listed in Table 1 with the respective amino acid change indicated. Site‐directed mutagenesis of the plasmids was confirmed by sequencing. Sequencing was performed using the dideoxy Sanger method by fluorescent automated sequencing on the ABI 3130xl genetic analyzer (Applied Biosystems, Waltham, MA, USA).

**Table 1:** List of primers used for insertion of C‐terminal HA‐tag and for SDM.

PrimerID	Description	Sequence (5′–3′)
LDLR_HA1F	HA‐tag first 5 amino acids	GAGGATGACGTGGCGTACCCATACGATGTTTGAACATCTGCCTGG
LDLR_HA1R		CCAGGCAGATGTTCAAACATCGTATGGGTACGCCACGTCATCCTC
LDLR_HA2F	HA‐tag last 4 amino acids	GTACCCATACGATGTTCCAGATTACGCTTGAACATCTGCCTGG
LDLR_HA2R		CCAGGCAGATGTTCAAGCGTAATCTGGAACATCGTATGGGTAC
LDLR_D445E_F	SDM_D445E: C>G	GAATCTACTGGTCTGAGCTGTCCCAGAGAATG
LDLR_D445E_R		CATTCTCTGGGACAGCTCAGACCAGTAGATTC
LDLR_D482H_F	SDM_D482H: G>C, C>T	CGACGGGCTGGCTGTGCATTGGATCCACAGCAACA
LDLR_D482H_R		TGTTGCTGTGGATCCAATGCACAGCCAGCCCGTCG
LDLR_C667F_F	SDM_C667F: G>T	GAGGAGTGAACTGGTTTGAGAGGACCACCC
LDLR_C667F_R		GGGTGGTCCTCTCAAACCAGTTCACTCCTC

### Cell culture, transfection, and treatments

HeLa cells were cultured in Dulbecco’s modified Eagle’s medium (DMEM; Invitrogen, Carlsbad, CA, USA) supplemented with 10% FBS (Invitrogen) and 100 U·mL^−1^ penicillin/streptomycin at 37 °C with 5% CO_2_. For immunostaining, cells were grown on sterile coverslips in 24‐well tissue culture plates and transient transfection was performed by FuGENE HD transfection reagent (Promega, Madison, WI, USA) as described previously 18. GFP‐H‐Ras plasmid 24 was used as a plasma membrane marker and cotransfected with HA‐tagged wild‐type or mutant plasmids. After 24 h of transfection, the cells were processed for staining and imaging as described previously 18.

Human embryonic kidney cells (HEK‐293T; ATCC, Manassas, VA, USA) were cultured in DMEM (Invitrogen) supplemented with 10% FBS (Invitrogen), and penicillin (10 U·mL^−1^) and streptomycin (100 μg·mL^−1^) at 37 °C with 5% CO_2_, and transfection was performed as described before 18. For transfection, cells were grown in 6‐well tissue culture plates and transfected with 1 μg plasmid DNA using FuGENE HD transfection reagent. The plasmid pGFPN2 was cotransfected along with the LDLR WT or mutants to control for transfection efficiency.

For blocking proteasome‐mediated degradation, serum‐starved cells were cultured in the presence of MG132 (10 µm), epoxomicin (100 nm), kifunensine (50 nm), or Eeyarestatin 1 (5 µm) for 16 h. For blocking lysosomal degradation, bafilomycin (200 nm) was added to the culture medium for 16 h. Cells were harvested for protein extraction after the treatments.

### Immunocytochemistry and imaging

Twenty‐four hours after transfection, HeLa cells grown on coverslips were processed for immunostaining as described previously 18, 25. Briefly, the coverslips were washed with PBS and fixed by methanol at −20 °C for 5 min. Fixed cells were washed in PBS and blocked in 1% BSA (Sigma‐Aldrich) in PBS for 30 min at room temperature. After blocking, the cells were incubated with mouse anti‐HA antibody and rabbit anti‐CANX antibodies, for 45 min at room temperature. Following incubation with the respective secondary antibodies for 45 min at room temperature, the coverslips were washed several times with PBS and mounted in immunofluor medium (ICN Biomedicals, Irvine, CA, USA). Confocal microscopy and imaging were performed with a Nikon Eclipse system (Nikon Instruments Inc., Tokyo, Japan) equipped with FITC and TRITC filters. Images were captured with a 100× oil immersion objective lens. All images presented are single sections in the z‐plane. Images were color enhanced and merged using imagej software 26.

### Cross‐linking of proteins, immunoprecipitation, and western blotting analysis

Forty‐eight hours after transfection, HEK‐293T cells were lysed in IP lysis buffer (Thermo Pierce, Rockford, IL, USA) containing protease inhibitors (Halt protease inhibitor cocktail; Thermo Fisher Scientific, Waltham, MA, USA) according to the manufacturer's instructions. Total protein concentration was determined by bicinchoninic acid protein assay (BCA kit; Thermo Pierce). For cross‐linking of proteins, the cells were washed twice with DPBS containing Ca^+^ and Mg^+^ (HyClone, Logan, UT, USA) and incubated with freshly prepared 1 mm dithiobis succinimidyl propionate (DSP; Sigma‐Aldrich), in DPBS for 30 min at room temperature. Unreacted DSP was quenched with 200 mm Tris, pH 7.5, for 15 min on ice, before proceeding with lysis in IP lysis buffer. HA‐tagged proteins were immunoprecipitated using anti‐HA agarose beads (Thermo Fisher Scientific) as described previously 19. For western blotting, the proteins were eluted from the beads by boiling in Laemmli sample buffer. The samples were then resolved on 7.5% SDS/PAGE gel followed by blotting onto nitrocellulose (Whatman Protran), or poly(vinylidene difluoride) (Thermo Fisher Scientific) membranes and probed with respective antibodies. Detection was performed using Enhanced Chemiluminescence Plus reagent (Thermo Pierce) and Typhoon FLA 9500 Imager (GE Healthcare Biosciences, Piscataway, NJ, USA). Densitometric analysis of the blots was performed by Image Studio Lite (Li‐COR), and graphs were generated by graphpad prism software (San Diego, CA, USA). Statistical significance was assessed by one‐way analysis of variance (ANOVA) followed by Dunnet's multiple comparison test.

### Endoglycosidase H sensitivity assay

For Endo H deglycosylation assay 18, the immunoprecipitates were denatured in 1× glycoprotein denaturation buffer (0.5% SDS and 1% β‐mercaptoethanol) for 5 min at 100 °C. The denatured proteins were then divided into two equal aliquots, which were incubated for 4 h at 37 °C in the presence or absence of 10 U of endoglycosidase H (Endo H; Sigma‐Aldrich). The digested samples were then resolved on a 7.5% SDS/PAGE gels and analyzed by western blotting as described above.

### Triton‐X solubility assay

Triton‐X solubility assay was carried out as described in 27. Briefly, cell pellets of HEK‐293T cells transiently expressing wt or mutant LDLR were lysed for 1 h at 4 °C in 240 μL 50 mm Tris/HCl, 150 mm NaCl, 1% Triton X‐100 (pH 7.6). Supernatant was collected as the detergent‐soluble fraction and the pellet as the insoluble fraction. The pellet was solubilized in an equal volume of SDS loading buffer.

### Quantitative real‐time PCR

Following transfection, total RNA was isolated from the transfected cells using Promega SV total RNA isolation system. Reverse transcription of total RNA was performed using Promega GoScript Reverse Transcriptase Kit, and quantitative real‐time PCR was performed on a QuantStudio Flex7 (Applied Biosystems) real‐time PCR machine as described in Ref. 19. TaqMan assays (Life Technologies, Carlsbad, CA, USA) for XBP‐1s (Hs03929085_g1) were used for analyzing ER stress and as internal reference control GAPDH (Hs02758991_g1) was used, according to the manufacturer's protocol. Gene expression was analyzed by comparative C*_t_* method using QuantStudio Real‐Time PCR software v 1.2. Statistical analysis was performed by Student's *t‐*test and one‐way ANOVA with *post hoc* Dunnet's multiple comparison test.

## Results

### D482H and C667F, but not D445E, missense mutations affected the trafficking and cell surface expression of LDLR

**Figure 1:**
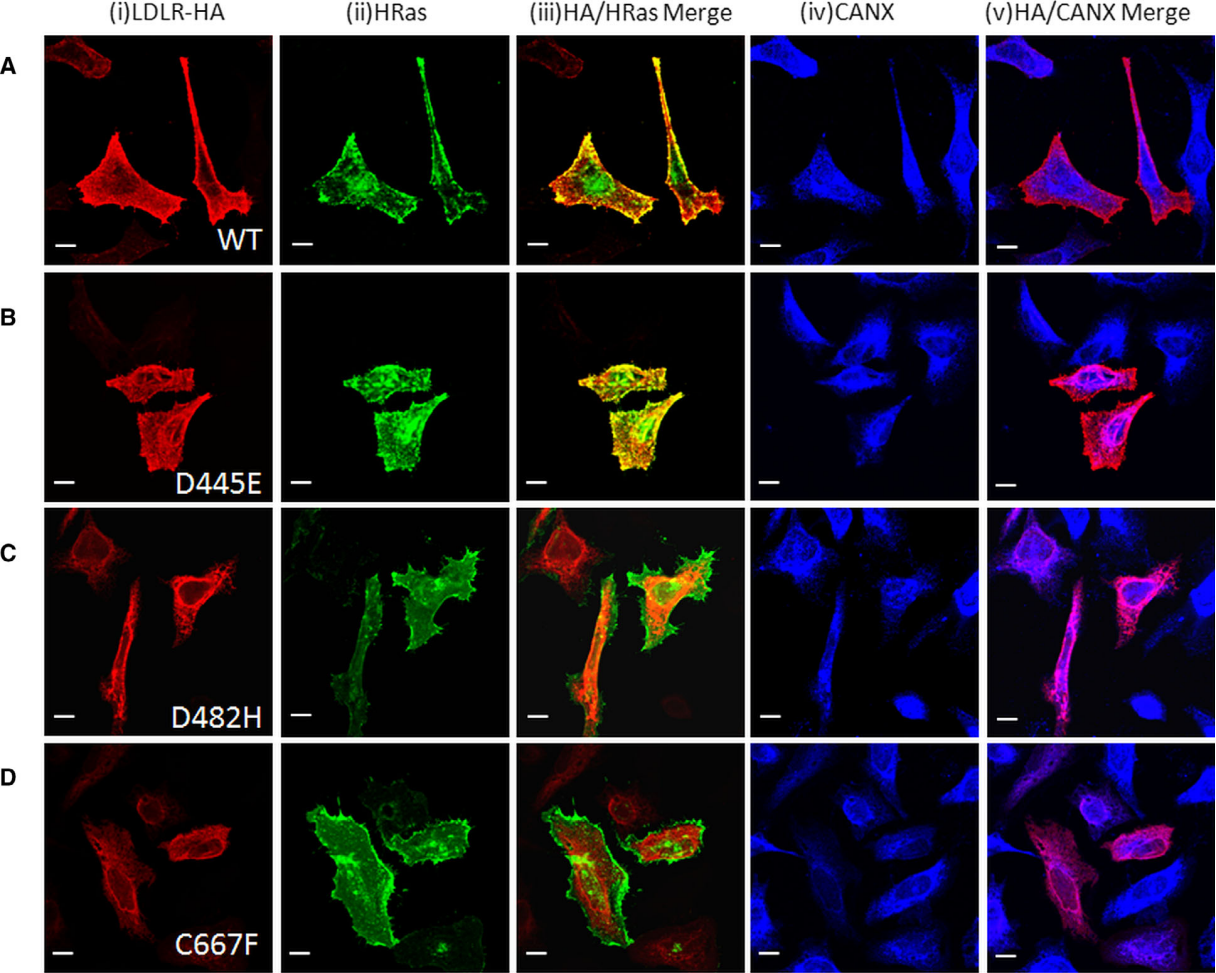
Comparison of intracellular localization of LDLR wild‐type and mutant variants: HeLa cells were transiently cotransfected with the indicated HA‐tagged LDLR plasmids (panels A‐D) and EGFP‐tagged H‐Ras and stained with anti‐HA antibodies and anti‐ CANX antibodies. Vertical panel (i) shows fluorescence staining pattern of HA from HeLa cells expressing the indicated LDLR‐HA plasmids, (ii) fluorescent signal from cells in the same field expressing GFP‐H‐Ras, (iii) merged image showing the extent of colocalization of both signals, (iv) shows fluorescent staining pattern of CANX in the same cells co‐expressing LDLR‐HA and GFP‐H‐Ras, and (v) indicates the merged images showing the extent of colocalization of LDLR with the ER marker CANX. Scale bar is 20 µm.

By site‐directed mutagenesis, we generated the three missense mutations (D445E, D482H, and C667F) found in FH patients and analyzed their impact at the cellular level. To determine the subcellular localization of the mutant receptors, HeLa cells were cotransfected with either the wild‐type or mutant constructs and the plasma membrane marker, H‐Ras tagged with EGFP. The wild‐type LDLR receptor was found to be localized to the plasma membrane as confirmed by its colocalization with GFP‐H‐Ras (Fig. 1, panels A(iii)). Though structural studies had predicted that, owing to the important function of the aspartate residue at position D445, even conserved amino acid changes would disrupt the structure and transport of the protein 28, the D445E mutant localized largely to the plasma membrane in similar manner to wild‐type (Fig. 1 panel B (iii)). The other two FH‐associated LDLR mutants, D482H and C667F, were found to be localized intracellularly, in a reticular and perinuclear pattern, which is characteristic for ER‐localized proteins (Fig. 1, panels C (i),D(i)). The ER localization of the mutants was confirmed by colocalization analysis with the ER marker, CANX. As apparent from panels A (iv,v),B (iv,v) in Fig. 1 , the localization pattern of the wild‐type and D445E mutant receptor was distinguishable from the localization of CANX. Other two mutants showed colocalization with CANX (Fig. 1, panels C (iv, v),D (iv,v)).

### The ER‐retained LDLR mutants are misfolded and have altered glycosylation profiles

**Figure 2:**
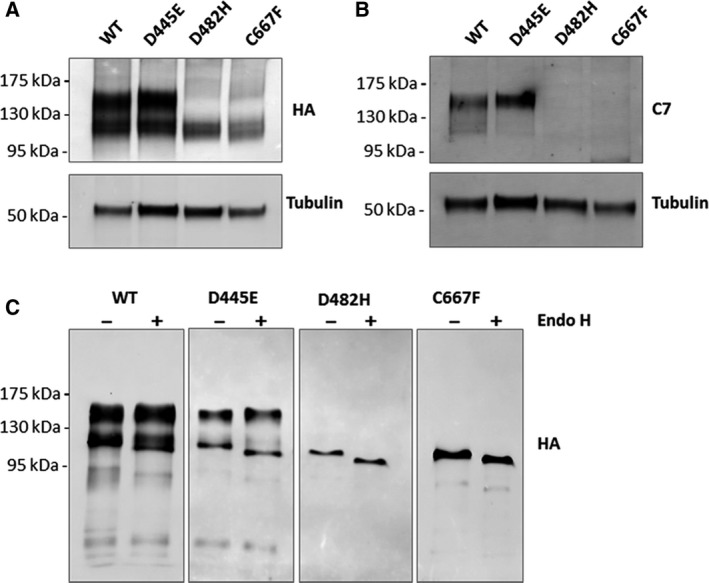
Analysis of the folding status of the LDLR mutants: Immunoblot analysis of total cell lysates from cells transiently transfected with HA‐tagged wild‐type or mutant LDLRs, under nonreducing conditions. (A) Immunoblots probed against HA antibody, showing difference in the migration of the mature (upper band) and precursor (lower band) forms of LDLR, among the wild‐type and mutants. (B) Immunoblots probed with LDLR C7 monoclonal antibody that specifically recognizes properly folded, mature LDLR. (C) Endo H susceptibility of the wild‐type LDLR and its mutants: HA‐tagged wild‐type LDLR or mutant variants were transiently expressed in HEK‐293T cells. HA‐tagged proteins were immunoprecipitated, treated with Endo H for 4 h at 37 °C (+) or left untreated for 4 h at 37 °C (−), and analyzed by immunoblotting with anti‐HA antibody. The mature form of the receptor was detectable in the immunoprecipitates from the wild‐type and D445E mutant and was resistant to Endo H digestion. ER forms of the wild‐type as well as the mutants were sensitive to Endo H treatment.

The mature form of LDLR contains both N‐linked and O‐linked glycosylation. Accordingly, in immunoblots, two bands of LDLR are detected: a faster migrating precursor form and a slower migrating fully glycosylated mature form. As anticipated, in immunoblots of total cell lysates overexpressing the wild‐type LDLR, both the precursor form (~ 120 kDa) and the mature form (~ 150 kDa) were observed, by anti‐HA antibody (Fig. 2A). In cell lysates overexpressing the LDLR D445E mutant also, the precursor and mature forms of the receptors were observed. In immunoblots of the mutants D482H and C667F, only the precursor form was observed (~ 120 kDa) and the mature receptor form was absent. To assess the folding status of the mutants, cell lysates from cells expressing either the wild‐type or mutants were analyzed by a conformation‐specific monoclonal antibody, LDLR‐C7, under nonreducing conditions. The LDLR‐C7 antibody binds to the correctly folded first cysteine‐rich repeat of the LDLR ligand‐binding domain and exclusively recognizes the native mature receptors 29. The C7 antibody was found to bind to the wild‐type LDLR and the D445E mutant, indicating that these receptors are correctly folded (Fig. 2B). The D482H and C667F mutants were not recognized by the C7 antibody suggesting that these mutants were not in the native conformation.

The glycosylation status of the mutant and wild‐type LDLR was determined by Endo H digestion of the immunoprecipitated proteins. Endo H specifically removes oligosaccharides of the high mannose and hybrid (pre‐Golgi) forms, but not complex carbohydrate structures attained in the Golgi. Figure 2C shows that the mutant LDLRs as well as of the precursor form of the wild‐type receptor were sensitive to Endo H digestion. As expected, the mature form of the wild‐type LDLR was resistant to Endo H treatment as it contains advanced glycosylation status attained in the Golgi. The N‐glycosylation profile of the mutants suggesting the absence of Golgi‐dependent glycosylation, and their colocalization with CANX establish that the mutant receptors are retained in the ER.

### ER‐retained LDLR mutants are soluble and exert ER stress

**Figure 3:**
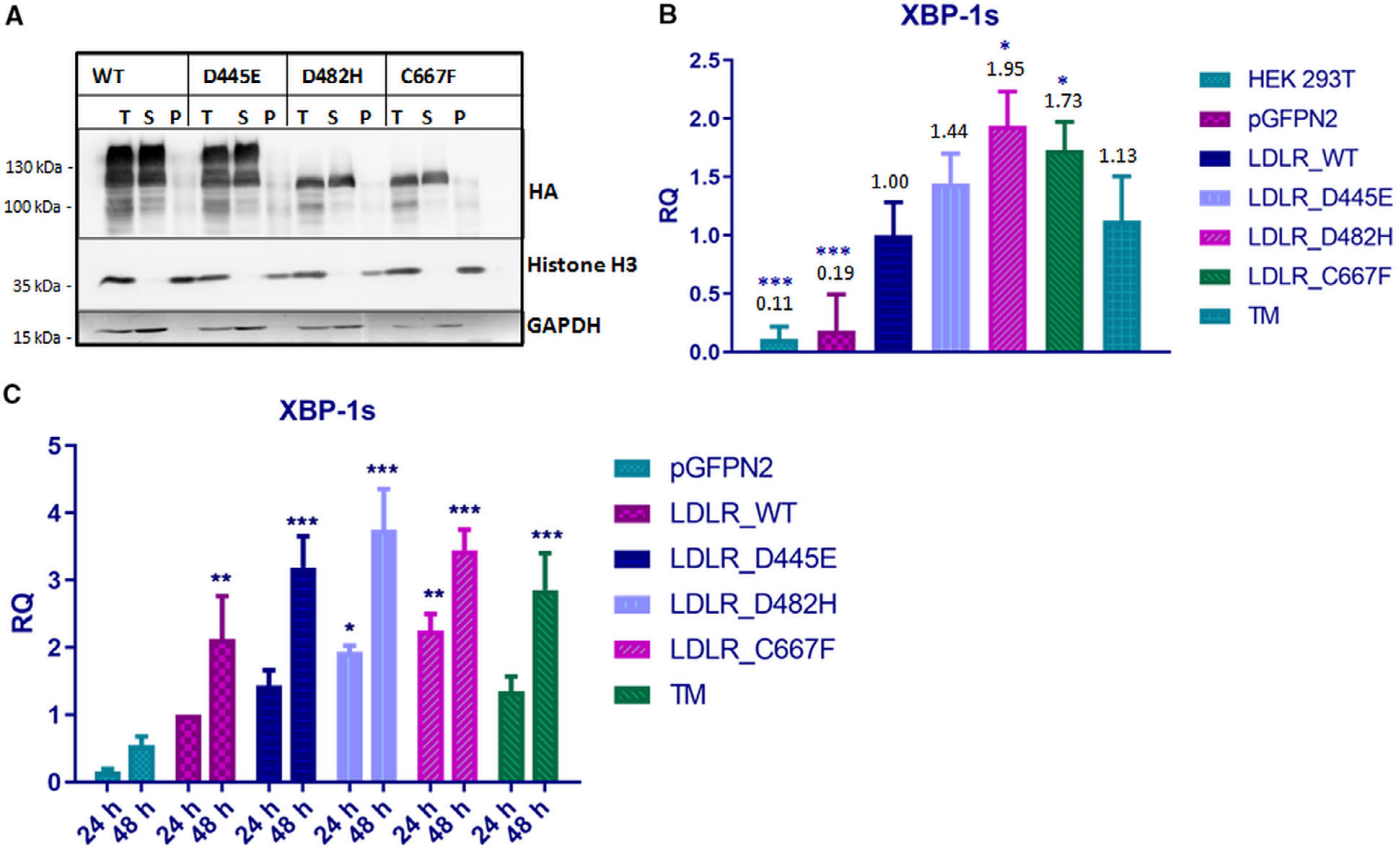
Analysis of aggregation states of LDLR WT and mutants and measurement of ER stress (A) Analysis of LDLR solubility in the nonionic detergent Triton X‐100. HEK‐293T cells were transiently transfected with the indicated plasmids. Cell extracts were prepared in lysis buffer supplemented with 1% Triton X‐100 and centrifuged at 4 °C at 20 000 *g* for 15 min. The total cell lysate (T), pellet (P), and supernatant (S) fractions were analyzed for the presence of respective LDLR proteins by western blot against HA. Histone H3 and GAPDH were used as controls for pellet and soluble fractions, respectively. The experiment was performed twice with similar results. (B) Induction of ER stress in HEK‐293T cells 48h post‐transfection with LDLR WT or mutants. Induction of ER stress was observed in cells expressing the mutants D482H and C667F, represented through elevated alternatively spliced XBP‐1 transcript levels, measured through quantitative PCR. TM‐treated cells were used as positive control for ER stress induction. The XBP‐1s mRNA levels of the LDLR WT at 48 h post‐transfection were set as 1.00. Fold changes in the mRNA expression of the mutants were expressed in relation to WT, and statistical significance was in comparison with WT. Error bars represent ± SEM of three independent experiments; (*) *P* ≤ 0.05; (**) *P* ≤ 0.01; (***) *P* ≤ 0.001; Student's *t*‐test. (C) Time‐dependant increase in the level of XBP‐1s in HEK‐293T cells expressing LDLR mutants. The XBP‐1s mRNA levels of the LDLR WT at 24 h post‐transfection were set as 1.00. Fold changes in the mRNA expression of the mutants were expressed in relation to WT. Statistical significance was tested against pGFPN2. Error bars represent ± SEM of three independent experiments; (*) *P* ≤ 0.05; (**) *P* ≤ 0.01; (***) *P* ≤ 0.001; one‐way ANOVA with Dunnet's *post hoc* multiple comparison test.

It was reported previously that retention of misfolded LDLR in ER results in the activation of unfolded stress response pathways 14. We examined the aggregation status of the LDLR mutants by Triton‐X solubility assay. The LDLR wild‐type and mutants were detected mostly in soluble fraction with a small proportion detected in the pellet (Fig. 3A). Accumulation of misfolded proteins in the ER leads to ER stress and the induction of UPR, which leads to the unconventional splicing of XBP‐1 mRNA 13. To analyze whether the ER retention of the LDLR mutants activates ER stress and UPR pathways, we measured the levels of spliced XBP‐1 mRNA (XBP‐1s). Tunicamycin (TM)‐treated cells were used as a positive control for ER stress induction. There was consistent elevation in XBP‐1s mRNA levels from D482H‐ and C667F‐expressing cells 48h post‐transfection, and it was found to be statistically significant from that of wild‐type (Fig. 3B). The XBP‐1s mRNA levels were found to be induced in D482H‐ and C667F‐expressing cells as early as 24 h post‐transfection (Fig. 3C). The fold change in the XBP‐1s mRNA levels in the D445E‐expressing cells was not significantly different from that of wild‐type‐expressing cells, confirming that this mutant is properly folded and transported out of ER as wild‐type.

### ER‐retained LDLR mutants show stronger association with folding chaperones

**Figure 4:**
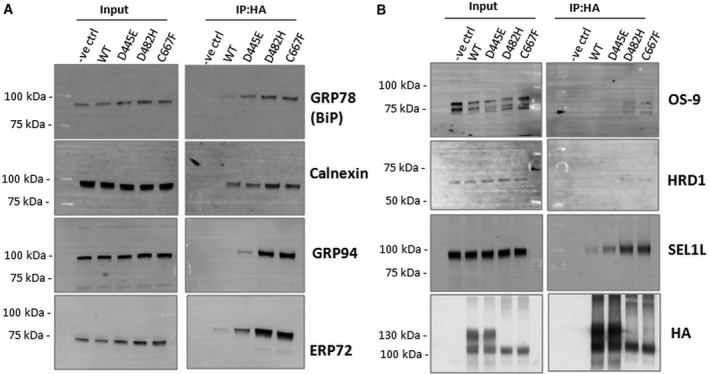
Association of LDLR mutants with ER chaperones and ERAD factors: (A) Cell lysates were prepared from transiently transfected cells expressing either wild‐type or mutant LDLR‐HA (D445E, D482H, and C667F). Cell lysates (200 µg) were subjected to Co‐IP using anti‐HA agarose beads. Western blot analysis of the immunoprecipitated complexes was performed using antibodies against GRP78 (BiP), CANX, GRP94, and ERP72. (B) The immunoblots from A were stripped and reprobed with antibodies against OS9, HRD1, SEL1L, and HA. The ER‐retained mutants show stronger interaction with the ERAD factors than the wild‐type and the D445E mutant. The experiments were performed twice for each construct with identical results.

A number of folding chaperones have been reported to be associated with wild‐type LDLR and involved in the ER retention of a class 2a LDLR variant, G544V 14. We asked whether ER retention of the LDLR variants analyzed in this study was favored by interaction with any of the previously known ER chaperones or folding enzymes. To study the interaction of LDLR mutants with ER quality control factors, cell lysates from HEK‐293T cells expressing the wild‐type or mutants were subjected to immunoprecipitation under nondenaturing conditions and probed with antibodies against known ER chaperones. To capture transient interactions, chemical cross‐linking by DSP was performed prior to cell lysis. The LDLR WT and all the three mutants were found to be associated with the heat‐shock family chaperones GRP78 (BiP), GRP94, the lectin chaperone CANX, and the folding enzyme ERP72 (Fig. 4A). Signals for the chaperones co‐immunoprecipitated with the wild‐type LDLR were very faint indicating transient and productive interactions. On the other hand, the signals of chaperones associated with the ER‐retained mutants D482H and C667F were several‐fold higher than that of the wild‐type indicating a stronger association of these mutants with the chaperones, likely leading to their ER retention. Intriguingly though the mutant D445E was found to traffic normally to the plasma membrane as wild‐type, it was found to associate with the chaperones with higher affinity than the wild‐type. This suggests that though this mutant is folding competent, it is probable that the folding efficiency is less than the wild‐type.

### LDLR mutants interact with HRD1/SEL1L complex and are degraded by the proteasomal pathway

We next examined the interaction of the LDLR WT and mutants with ERAD components OS‐9, SEL1L, and HRD1. All the three ERAD factors co‐immunoprecipitated with the ER‐retained mutants with greater affinity than the wild‐type or D445E mutant (Fig. 4B). This suggested that the ER‐retained LDLR mutants are degraded by the HRD1/SEL1L‐mediated ERAD. We analyzed the involvement of the proteasome in the degradation of these mutants. We used inhibitors that block various steps in the recognition and targeting of ERAD clients for degradation.

**Figure 5:**
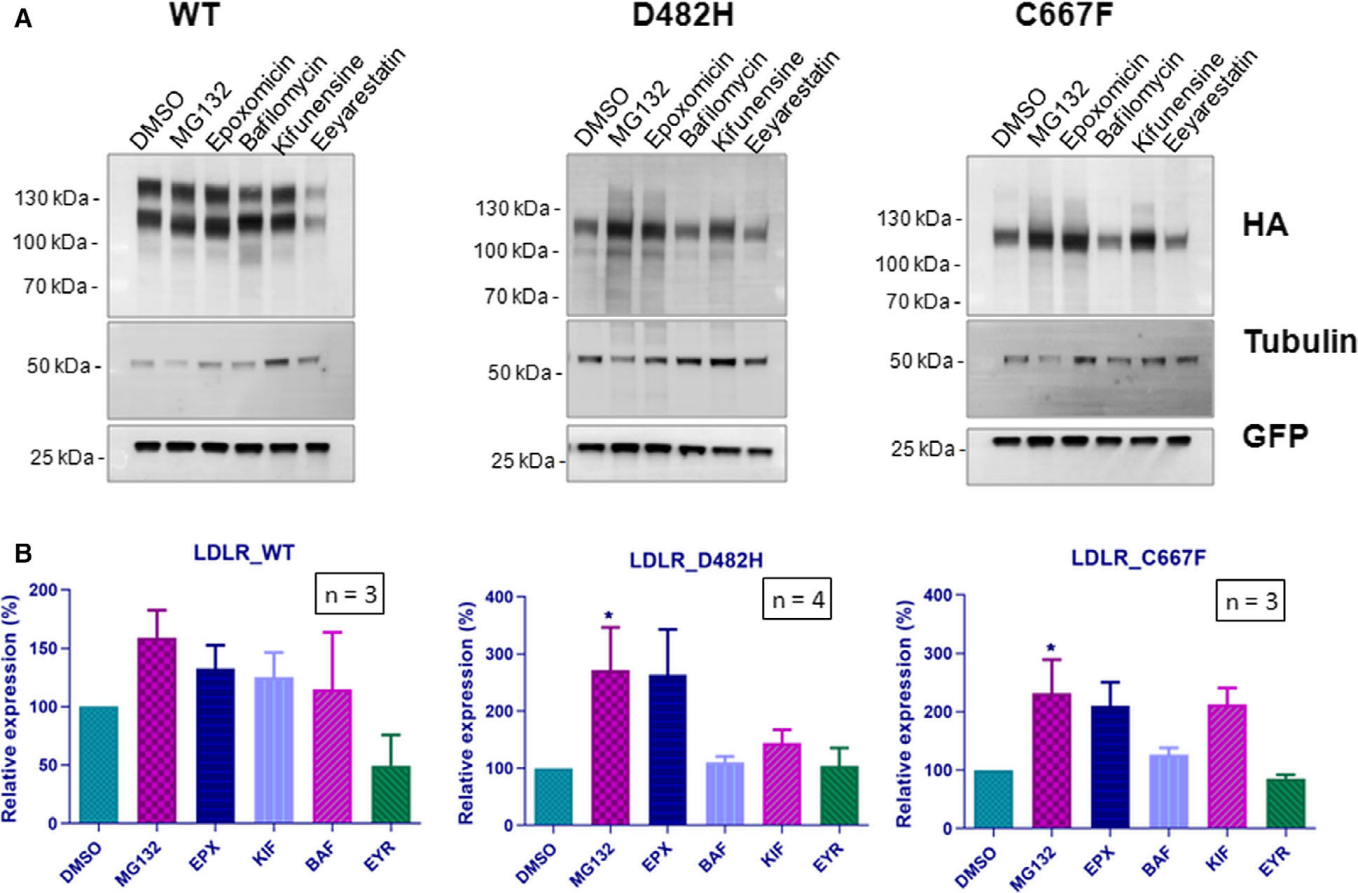
Accumulation of ER‐retained LDLR mutants in response to proteasomal inhibition: (A) HeLa cells transiently expressing the LDLR WT or the mutants D482H or C667F were treated with a panel of inhibitors targeting the proteasome (MG132, epoxomicin)/lysosome (bafilomycin) or specific steps in the ERAD pathway (kifunensine, Eeyarestatin 1). Total cell lysates were analyzed by immunoblotting against HA. Tubulin was used as loading control, and GFP was used as transfection control. Relative amounts of respective proteins remaining at the indicated time points were quantified and normalized to tubulin levels. The experiments were performed thrice with identical results. (B) Graph representing the relative mean densities of vehicle (DMSO)‐ or inhibitor‐treated wild‐type LDLR and the two mutants. Error bars represent SEM from at least *n* = 3 independent experiments. Statistical significance was assessed by one‐way ANOVA and Dunnet's *post hoc* multiple comparison test.

Inhibitors of proteasome MG132 and epoxomicin had a striking effect in stabilizing the steady‐state levels of ER‐retained variants D482H and C667F (Fig. 5A,B) while the lysosomal inhibitor bafilomycin had no effect on the steady‐state levels of the mutants. Kifunensine is an inhibitor of ER mannosidase I and interferes with early substrate recognition for ERAD 30. The expression levels of mutants D482H and C667F were found to be enhanced in response to treatment with kifunensine also. An inhibitor of p97 ATPase, Eeyarestatin I which is known to prevent the retrotranslocation of misfolded substrates 31, did not have any effect on the steady‐state levels of the mutants. Though expression level of exogenously expressed wild‐type LDLR receptor was reported to be unaffected by proteasome inhibition 8, our results indicated that treatment with the proteasome inhibitors MG132 and epoxomicin led to the accumulation of the wild‐type receptor in HeLa cells. Together, our results indicate that the ER‐retained LDLR mutants D482H and C667F are subject to degradation by HRD1/SEL1L‐mediated proteasomal degradation.

## Discussion

In this report, we have analyzed the cellular consequences of three, missense mutations in the LDLR gene associated with FH. We provide evidence that two of these mutations lead to a ER retention and proteasome‐mediated degradation of the mutant receptors.

The disease‐causing mutations p.D445E and p.D482H affect conserved aspartate residues in the β‐propeller domain. The C667F mutation affects a highly conserved cysteine residue in the extracellular EGF‐like 3 domain of the LDLR protein. These residues are structurally conserved in the closely related VLDLR (D487, D521, C706), and substitutions in the corresponding positions lead to misfolding and ER retention of the mutants 18. It was implied from the crystal structure of LDLR that, in the consensus repeat motifs (Tyr‐Trp‐Thr‐Asp) of the β‐propeller domain, the Asp residues anchor adjacent blades of the β‐propeller, stabilizing the structure 28. It was predicted that since both the carboxylated oxygen of Asp residues are acceptors of structurally conserved hydrogen bonds, even conserved substitutions such as D445E can disrupt the structure and eventually, transport of the protein 28. We have observed that in HeLa cells expressing the D445E mutant, the localization and N‐glycosylation pattern of the mutant were identical to that of wild‐type LDLR suggesting that the conserved amino acid change did not result in any folding and trafficking defect. It can be assumed that while it is possible that a substitution of glutamic acid at this position can exert structural restraint, it can still retain hydrogen bonding interactions and may not critically alter the structural stability of the protein during folding. The amino acid substitutions, D482H and C667F, lead to ER retention of the mutant variants and affected transport to the cell surface, likely due to misfolding. To analyze the folding status of the ER‐retained mutants, we performed immunoblot analysis of the wild‐type and mutants under nonreducing conditions against a conformation‐specific LDLR antibody (C7 mAb). The C7 mAb recognizes the first of seven cysteine‐rich repeats, of the N‐terminal ligand binding of LDLR, if the native disulfide bonds are intact 29. The wild‐type and the D445E mutant, but not the D482H and C667 mutants, were able to bind to the C7 antibody, confirming that the D445E mutant has no folding defect. The C7 epitope located on the most amino‐terminal domain is reported to acquire its native conformation later in the productive folding cycle 32. Thus, the D482H and C667F mutants were sequestered in the ER due to misfolding.

From this study, it is evident that the D445E mutant is transported to the cell surface and is not a class II mutant. Some studies have indicated not all cosegregating LDLR variants reported may actually be the mutation responsible for the observed clinical phenotype 33, 34. Mutations that lead to premature stop codons, frameshift, or large deletion/rearrangements generally result in no apparent protein production or a truncated dysfunctional protein. Similarly, missense mutations that alter a conserved amino acid at a critical position typically result in a defective LDL receptor protein. These classes of mutations are more probably causative if identified in a clinically diagnosed patient. In contrast, conservative missense variations, silent mutations, and noncoding variations may not be pathogenic and necessitate further functional studies 7.

Previously, it has been reported that retention of LDLR mutants in the ER induces ER stress and activates the UPR pathways 14. ER‐retained VLDLR mutants were found to have longer cellular half life than the wild‐type, were aggregation‐prone, and induced ER stress 19. Our analysis revealed that the LDLR mutants are soluble and are not aggregation‐prone. We next analyzed the ER stress levels of the cells expressing the LDLR WT and mutants. We observed that the expression of the mutants D482H and C667F induced the expression of spliced XBP‐1 mRNA, indicating activation of ER stress. It was indicated that therapeutic strategies in heterozygous FH patients that utilize agents that enhance LDLR expression could induce ER stress in the cells due to enhanced expression of the misfolded LDLR as well. Chemical chaperones that can assist folding of mutant proteins could be an alternative strategy for these types of mutations, and chemical chaperones like glycerol and 4‐PBA have been reported to be able to restore functionality of some of the LDLR class 2 receptors in a mutation‐specific manner 35

To analyze whether the ER retention of the LDLR mutants was favored by interactions with any known folding chaperones, we performed co‐immunoprecipitation (Co‐IP). It was observed that GRP78 (BiP), CANX, GRP94, and ERP72 formed a stronger association with the ER‐retained mutants than with the wild‐type and the D445E mutant. The two major chaperone systems in the ER are the CANX /calreticulin system and the GRP78/GRP94 system 36. The lectin‐like chaperones CANX and calreticulin recognize the presence of both monoglucosylated N‐linked glycans and unfolded regions on nascent glycoproteins. Glucose‐regulated proteins GRP78 and GRP94 are chaperones belonging to the heat‐shock family 36. As a chaperone, GRP78 recognizes and binds to unfolded regions on proteins containing hydrophobic residues. GRP78 exists in a multiprotein complex with a large set of ER molecular chaperones, which include GRP94, PDI, ERp72 37. ERP72 is a folding catalyst belonging to the thiol oxidoreductases family. The expression of all the four chaperones that interact with the mutants has been reported to increase under ER stress 38. All these chaperones were previously reported to interact with a class II mutant G544V but not with the wild‐type LDLR. We observed faint signals of these chaperones in co‐immunoprecipitates of wild‐type as well, probably representing transient and productive interactions. It is likely that these are general ER components participating in the ER retention of LDLR mutants.

Terminally misfolded proteins are diverted for degradation to eliminate ER stress and restore homeostasis. It was shown previously that proteasomal degradation is the primary pathway for degradation of LDLR class II mutants and inhibition of proteasome function restored trafficking of these mutants 8. However, the molecular components assisting the degradation of the mutants were not studied. HRD1/SEL1L complex is a principal component of mammalian ERAD, and we have recently reported that SEL1L participates in the degradation of VLDLR mutants 19. By Co‐IP, we found that the D482H and C667F mutants form stable associations with HRD1 and its partners SEL1L and OS9. OS9 is a lectin chaperone in the ER which is reported to extract misfolded proteins from GRP94 and deliver to HRD1‐SEL1L complex for degradation 39. Inhibition of proteasome activity resulted in the accumulation of the ER‐retained mutants, but had no effect on folding as indicated by the absence of the mature form the receptor in immunoblots. Also, kifunensine, which inhibits ER mannosidase I and thus prevent early substrate recognition, also had a stabilizing effect on the mutants, confirming that the ER‐retained LDLR mutants are subject to degradation by HRD1/SEL1L‐mediated proteasomal degradation.

## Conclusions

In conclusion, our results indicate that ER retention and proteasomal degradation are involved in the loss of function of two of the LDLR missense mutations studied here. Our study confirms that ER retention of class II mutants of LDLR induces ER stress and involves association with folding chaperones CANX, GRP78, GRP94, and ERP72. We also report the involvement of ERAD components OS9, HRD1, and SEL1L in the degradation of LDLR mutants. As opposed to structure‐based predictions, a conserved amino acid substitution in a crucial domain of LDLR did not interfere with the folding and trafficking of the mutant thus emphasizing the importance *in vitro* assays in complementing structure‐based pathogenicity predictions.

## Conflict of interest

The authors declare no conflict of interest.

## Author contributions

BRA and PK were responsible for the project conception and design of the experiments. PK, AJ, and BKA carried out the experiments. All the authors contributed to data analysis and interpretation. PK drafted the manuscript, and BRA, LA, and AJ took part in editing the manuscript. All of the authors read and approved the final manuscript.
